# Whole-exome sequencing identifies susceptibility genes and pathways for idiopathic pulmonary fibrosis in the Chinese population

**DOI:** 10.1038/s41598-020-80944-4

**Published:** 2021-01-14

**Authors:** Chuling Fang, Hui Huang, Yujia Feng, Qian Zhang, Na Wang, Xiaoyan Jing, Jian Guo, Martin Ferianc, Zuojun Xu

**Affiliations:** 1 Department of Respiratory and Critical Medicine, Peking Union Medical College Hospital, Chinese Academy of Medical Sciences and Peking Union Medical College, Beijing, China; 2 The Bioinformatics Department, Digital China Health Technologies Co., Ltd, Beijing, China; 3 Electronic and Electrical Engineering Department, University College London, London, UK

**Keywords:** Disease genetics, Respiratory tract diseases

## Abstract

Genetic factors play a role in the risk of idiopathic pulmonary fibrosis (IPF). Specifically, *MUC5B* rs35705950 non-risk alleles and immunologic aberrations were associated with the IPF’s progression. However, rare genetic variants have not been systematically investigated in Chinese IPF patients. In this study, we aimed to improve understanding of the genetic architecture of IPF in the Chinese population and to assess whether rare protein-coding variants in the immunity pathway genes are enriched in the IPF patients with non-risk alleles at rs35705950. A case–control exome-wide study including 110 IPF patients and 60 matched healthy controls was conducted. rs35705950 was genotyped by Sanger sequencing. To identify genes enriched in IPF, gene-based association analyses were performed. Identified genes were included for further pathway analyses using gene ontology (GO) and Kyoto Encyclopedia of Genes and Genomes (KEGG). Associations between rs35705950 and genes enriched in the immunity pathway were also tested. 226 genes that were enriched with deleterious variants were identified in IPF patients. Out of them, 36 genes were significantly enriched in GO and KEGG pathways in the IPF. Pathway analyses implicated that these genes were involved in the immune response and cell adhesion. Rare protein-altering variants in genes related to the immunity pathway did not significantly differ between patients with a *MUC5B* risk allele and individuals without risk allele. We drafted a comprehensive mutational landscape of rare protein-coding variants in the Chinese IPF and identified genes related to immune response and cell adhesion. These results partially explain changes in gene expression involved in the immunity/inflammatory pathways in IPF patients.

## Introduction

Idiopathic pulmonary fibrosis (IPF), one of the common types of idiopathic interstitial pneumonia (IIP), is characterized by progressive fibrosis of unknown aetiology^1^. IPF occurs primarily in men aged from 50 to 70, with a median survival of 2–3 years^2^. Two approved therapies, pirfenidone and nintedanib, appear to decelerate disease progression and have a mortality benefit^3,4^. Its pathogenic mechanisms have not been elucidated and no medical treatment to date has been found to cure IPF except lung transplantation^5^.

Genetic variants, rare and common, contribute to the susceptibility of IPF in both sporadic and familial cases^6–8^. Previous studies in patients with familial pulmonary fibrosis (FPF) or sporadic IPF have identified common variants (minor allele frequency (MAF) > 5%). A single nucleotide polymorphism (SNP) rs35705950 in the promoter region of the *MUC5B* gene demonstrated to be strongly associated with the IPF and familial interstitial pneumonia (FIP) in one genome-wide linkage analysis^9^ and the association was validated in multiple different cohorts^10–16^. Additionally, other common variants in several genes were also found to be associated with the disease, by genome-wide approaches, such as DNA repair-related genes (*TERT*^17^, *TERC*, *OBFC1*), host defense-related genes (*ATP11A*, *TOLLIP*), cell–cell adhesion-related genes (*DSP*, *DPP9*), profibrotic signaling pathway-related genes (*AKAP13*), *FAM13A* and *SPPL2C*^10,11,18–20^. In addition to the common variants, rare variants were also reported to be involved in two main pathways in IPF, including telomere maintenance and surfactant metabolism. Rare variants in multiple different telomerase related genes (*TERT*, *TERC*, *RTEL1* and *PARN*) have been associated with both familial^21–25^ and sporadic IPF^10,26–28^. Two genes involved in encoding surfactant proteins A and C (*SFTPA2* and *SFTPC*) have also been related to the IPF in sporadic studies^29,30^. However, these risk variants generally explain a relatively small proportion of IPF’s heritability; except for rs35705950.

Whole-exome sequencing (WES) has become an increasingly popular approach to identify rare alleles with direct functional consequences on protein products. However, WES has not been extended to the studies on sporadic IPF in the Chinese population. Besides, previous research on the IPF subjects indicated that *MUC5B* risk allele was associated with longer survival^31^ and aberrant immunity was related to IPF progression^10,11,32–37^. In this study, we performed WES to identify genes carrying excessive rare deleterious variants, investigated their aggregate effects by pathway analyses and assessed if candidate genes involved in the immunity pathway were enriched in IPF patients with non-risk alleles at rs35705950.

## Results

### Subjects’ characteristics and sequencing data

A total of 110 IPF patients and 60 matched controls were included in the study. Baseline characteristics of 170 participants were summarized in Table 1. Cases and controls were similar in age, gender proportion and smoking status due to matching. No significant difference was found in BMI between the two groups (*P* = 0.701).

**Table 1:** Baseline characteristics of the included subjects.

Characteristics	IPF cases (n = 110)	Controls (n = 60)	*P* value
Age	63.49 ± 8.23	63.43 ± 8.36	0.965
Male (%)	101 (91.8)	54 (90.0)	0.196
BMI (kg/m^2^)	24.08 ± 2.53	24.23 ± 2.48	0.701
Smoking status			0.741
Former/current	42 (38.2)	21 (35.0)	
Never	68 (61.8)	39 (65.0)	
Clinical manifestation			
Cough	107 (97.3)	0	
Dyspnea	80 (72.7)	0	
Finger clubbing	52 (47.3)	0	
Velcro rales	101 (91.8)	0	
PFT			
FVC (% predicted)	72.39 ± 15.36		
DLco (% predicted)	46.45 ± 13.03		

All values are reported as mean ± SD = standard deviation or percentage. IPF = idiopathic pulmonary fibrosis; PFT = pulmonary function test; FVC % pred = percent predicted forced vital capacity; DLCO% pred = percent predicted diffusion capacity for carbon monoxide. P values were from the Student’s *t* test for continuous variables or from the Chi-square test for categorical variables.

Average sequencing depth of targeted exome regions in all tested samples was 110 × . The mean proportion of targets that were covered with at least 10 × was 99%.

## Rare deleterious variants in IPF and candidate genes enriched with deleterious variants

After multi-step filtering (Supplementary Fig. S1), a total of 10,333 deleterious variants with MAF < 0.01 were identified in the IPF group, including a large proportion of nonsynonymous variants (n = 7093) and a small number of frameshift indels (n = 2136), stop-gain (n = 1058) and stop-loss (n = 46). After gene-based association analysis for all the above-mentioned variants by SKAT, we identified 226 genes that were enriched with rare, deleterious variants in the Chinese IPF samples (all P-FDR < 0.05) (Supplementary Table S1). Given the complexity of HLA, the association of HLA genes selected from SKAT with IPF was further determined by another collapsing analysis. The results showed that except *HLA-G*, other HLA genes were significantly associated with IPF (P-FDR < 0.05), and P value of *HLA-G* was equal to 0.05, which was at the critical level. The above HLA genes were included in the subsequent analysis (GO and KEGG), because the objective of this study was to find genes and pathways that might be related to IPF in a more conservative way, and to avoid missing genes potentially associated with the IPF. Finally, a total of 226 candidate genes were included in subsequent analyses.

## Enrichment and pathway discovery

**Figure 1:**
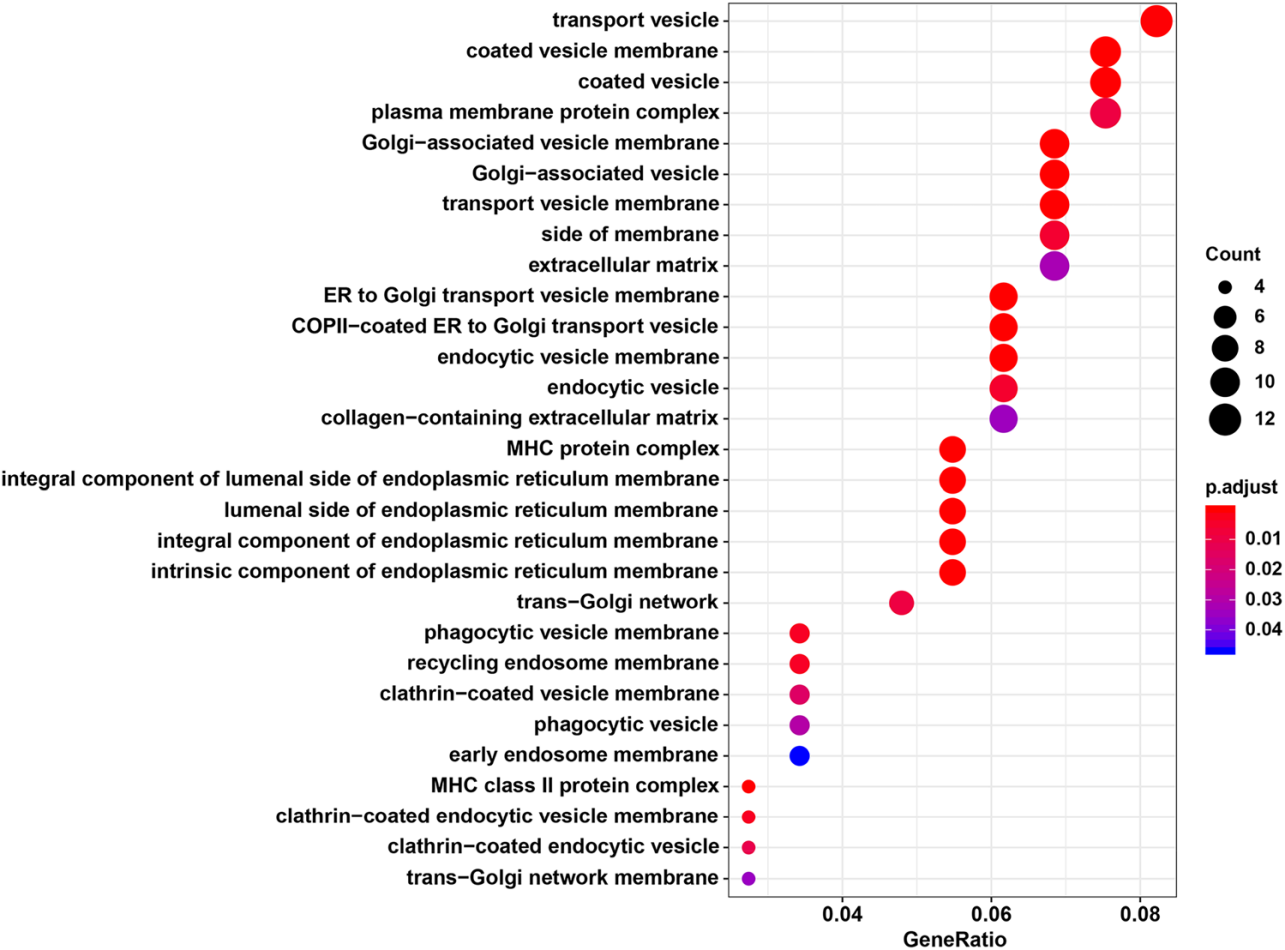
Cellular components terms of gene ontology analysis of candidate genes. The X-axis represents the ratio of genes enriched in the pathway to the total enriched genes, and the Y-axis represents the terms of enriched cellular components. The dot size represents the number of genes enriched in the pathway, and the color represents the P value. P value was adjusted by Benjamini–Hochberg false discovery rate.

GO and KEGG pathway enrichment analyses were performed with the input consisting of 226 genes. Figure 1 shows that, for cellular components, mutated genes were mainly involved in vesicle, membrane, extracellular matrix and MHC protein complex (all P.adjusted < 0.05). In terms of biological process, ‘Interferon-gamma-mediated signaling pathway’ (P.adjusted = 7.852 × 10^–5^) and ‘Antigen processing and presentation of peptide antigen’ (P.adjusted = 2.190 × 10^–3^) showed the most significance, which can be observed in Table 2. As for the molecular function, candidate genes were significantly enriched in ‘peptide antigen binding’ (P.adjusted = 5.958 × 10^–7^) and ‘amide binding’ (P.adjusted = 3.599 × 10^–3^), similarly seen in Table 2. Additionally, KEGG pathways of the mutated genes included mainly ‘antigen processing and presentation’ (P.adjusted = 5.805 × 10^–8^), ‘phagosome’ (P.adjusted = 6.283 × 10^–5^), ‘cell adhesion molecules (CAMs)’ (P.adjusted = 3.585 × 10^–4^), ‘natural killer cell mediated cytotoxicity’ (P.adjusted = 5.858 × 10^–3^) and ‘Th1 and Th2 cell differentiation’ (P.adjusted = 0.042), seen in Table 3.

**Table 2:** Biological process and molecular function of gene ontology (GO) analysis of candidate genes.

	P value	Genes
***GO-biological process***		
Interferon-gamma-mediated signaling pathway	7.852 × 10^–5^	*OTOP1, HLA-H, HLA-A, HLA-B, HLA-DRB5, HLA-DRB1, HLA-DQA1, HLA-DQB1, HLA-G*
Antigen processing and presentation of peptide antigen	2.190 × 10^–3^	*HLA-H, HLA-A, HLA-B, HLA-DRB5, HLA-DRB1, HLA-DQA1, HLA-DQB1, SEC22B, ERAP1, HLA-G*
Cellular response to interferon-gamma	2.190 × 10^–3^	*OTOP1, HLA-H, HLA-A, HLA-B, HLA-DRB5, HLA-DRB1, HLA-DQA1, HLA-DQB1, HLA-G*
Response to interferon-gamma	2.927 × 10^–3^	*OTOP1, HLA-H, HLA-A, HLA-B, HLA-DRB5, HLA-DRB1, HLA-DQA1, HLA-DQB1, HLA-G*
Antigen processing and presentation of exogenous peptide antigen	2.927 × 10^–3^	*HLA-H, HLA-A, HLA-B, HLA-DRB5, HLA-DRB1, HLA-DQA1, HLA-DQB1, SEC22B, HLA-G*
Antigen processing and presentation	2.927 × 10^–3^	*HLA-H, HLA-A, HLA-B, HLA-DRB5, HLA-DRB1, HLA-DQA1, HLA-DQB1, SEC22B, ERAP1, HLA-G*
Antigen processing and presentation of exogenous antigen	2.927 × 10^–3^	*HLA-H, HLA-A, HLA-B, HLA-DRB5, HLA-DRB1, HLA-DQA1, HLA-DQB1, SEC22B, HLA-G*
Antigen processing and presentation of peptide antigen via MHC class I	0.024	*HLA-H, HLA-A, HLA-B, SEC22B, ERAP1, HLA-G*
***GO-molecular function***		
Peptide antigen binding	5.958 × 10^–7^	*HLA-A, HLA-B, HLA-DRB5, HLA-DRB1, HLA-DQA1, HLA-DQB1, HLA-G*
Amide binding	3.599 × 10^–3^	*FTCDNL1, HLA-A, HLA-B, HLA-DRB5, HLA-DRB1, HLA-DQA1, HLA-DQB1, FOLH1, CHRNA7, HLA-G*
Peptide binding	3.599 × 10^–3^	*HLA-A, HLA-B, HLA-DRB5, HLA-DRB1, HLA-DQA1, HLA-DQB1, FOLH1, CHRNA7, HLA-G*
Antigen binding	9.998 × 10^–3^	*HLA-A, HLA-B, HLA-DRB5, HLA-DRB1, HLA-DQA1, HLA-DQB1, HLA-G*

P values were from gene ontology (GO) analysis and adjusted by Benjamini–Hochberg false discovery rate.

**Table 3:** Kyoto Encyclopedia of Genes and Genomes (KEGG) pathway analysis and candidate genes.

Pathway	P value	Genes
Antigen processing and presentation	5.805 × 10^–8^	*HSPA6, KIR2DL1, HLA-A, HLA-B, HLA-DRB5, HLA-DRB1, HLA-DQA1, HLA-DQB1, KIR2DS4, HLA-G*
Phagosome	6.283 × 10^–5^	*HLA-A, HLA-B, HLA-DRB5, HLA-DRB1, HLA-DQA1, HLA-DQB1, SEC22B, PLA2R1, HLA-G*
Cell adhesion molecules (CAMs)	3.585 × 10^–4^	*HLA-A, HLA-B, HLA-DRB5, HLA-DRB1, HLA-DQA1, HLA-DQB1, ITGA6, HLA-G*
Natural killer cell mediated cytotoxicity	5.858 × 10^–3^	*KIR2DL1, HLA-A, HLA-B, PLCG2, KIR2DS4, HLA-G*
Th1 and Th2 cell differentiation	0.042	*HLA-DRB5, HLA-DRB1, HLA-DQA1, HLA-DQB1*

P values were from Kyoto Encyclopedia of Genes and Genomes (KEGG) pathway analysis and P values were adjusted by Benjamini–Hochberg false discovery rate.

## Mutational landscape of genes enriched in GO and KEGG pathways in IPF

**Figure 2:**
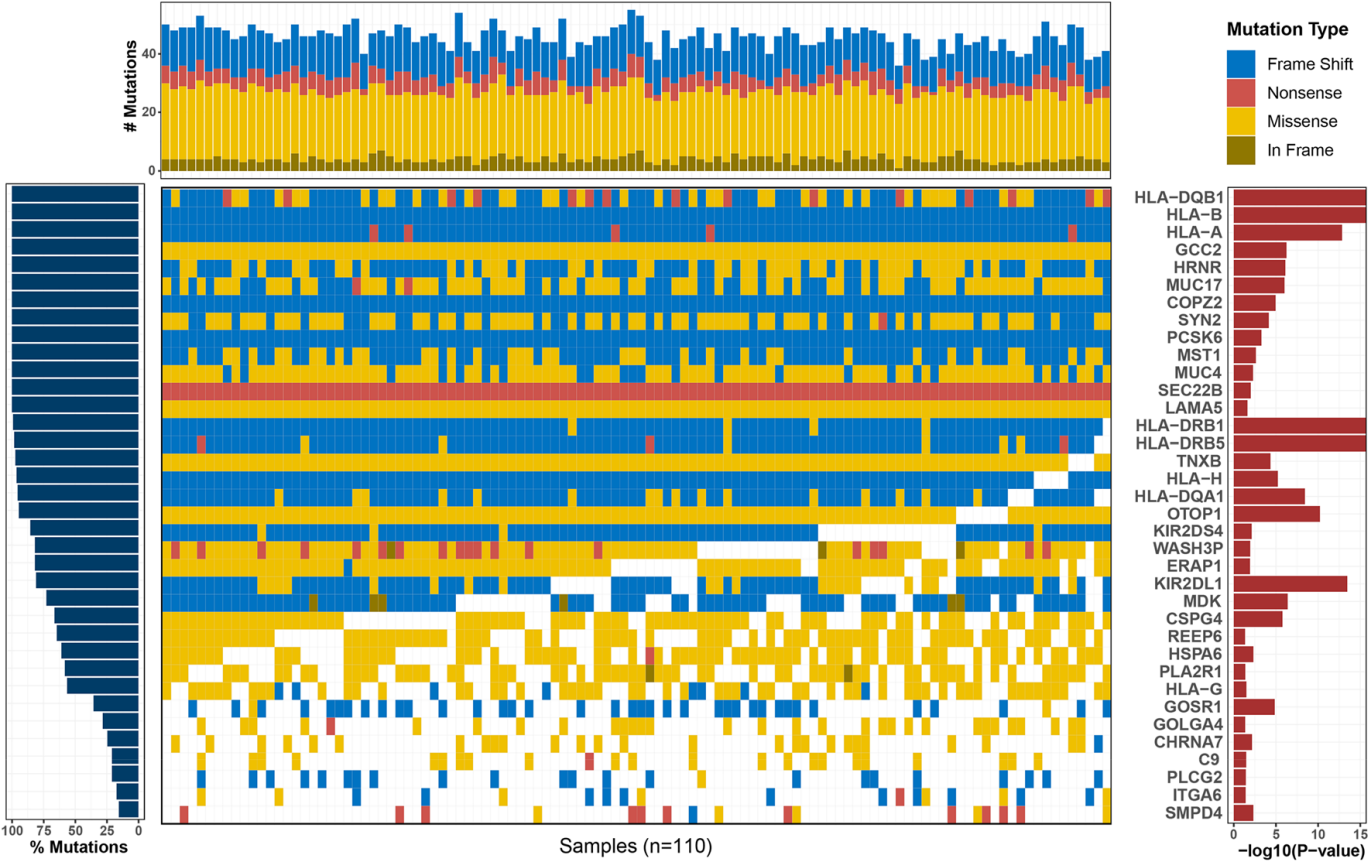
Mutational landscape of 36 candidate genes enriched in GO and KEGG pathways in the IPF samples. Mutation burdens are displayed on the X-axis panel (top), and cohort mutation percentage by a gene is displayed on the Y-axis panel (left). P value from SKAT of each gene is displayed on the Y-axis panel (right). Displayed genes are significantly enriched in the IPF samples. Each column represents an individual sample. Coloring of patient-gene intersection grid indicates mutation type (top legend).

A total of 36 genes were significantly enriched in GO and KEGG pathways in IPF samples, seen in Fig. 2. All IPF samples (100%) had rare deleterious variants in *HLA-DQB1*, *HLA-B*, *HLA-A*, *GCC2*, *HRNR*, *MUC17*, *COPZ2*, *SYN2*, *PCSK6*, *MST1*, *MUC4*, *SEC22B* and *LAMA5*.

## Associations between *MUC5B* risk allele and genes enriched in the immunity pathway

Table 4 presents the associations between *MUC5B* rs35705950 and immunity-related genes with rare deleterious variations. No genes were found to be significantly associated with *MUC5B* risk allele (all P > 0.05).

**Table 4:** Associations between *MUC5B* risk allele and mutated genes involved in the immunity pathway.

	IPF patients without *MUC5B* risk allele (n = 93)	IPF patients with *MUC5B* risk allele (n = 17)	OR (95% CI)	*P* value
*HLA-DQB1*	93 (100.0)	17 (100.0)	–	–
*HLA-B*	93 (100.0)	17 (100.0)	–	–
*HLA-A*	93 (100.0)	17 (100.0)	–	–
*SEC22B*	93 (100.0)	17 (100.0)	–	–
*HLA-DRB1*	93 (100.0)	16 (94.1)	NA	1.000
*HLA-DRB5*	92 (98.9)	16 (94.1)	0.17 (0.01–2.92)	0.224
*HLA-H*	89 (95.7)	17 (100.0)	NA	0.999
*HLA-DQA1*	89 (95.7)	16 (94.1)	0.72 (0.08–6.86)	0.774
*KIR2DS4*	78 (83.9)	16 (94.1)	3.08 (0.38–24.99)	0.293
*ERAP1*	75 (80.6)	15 (88.2)	1.80 (0.38–8.59)	0.461
*KIR2DL1*	73 (78.5)	16 (94.1)	4.38 (0.55–35.09)	0.164
*HSPA6*	54 (58.1)	13 (76.5)	2.35 (0.71–7.75)	0.161
*PLA2R1*	56 (60.2)	8 (47.1)	0.59 (0.21–1.66)	0.315
*HLA-G*	50 (53.8)	12 (70.6)	2.06 (0.67–6.33)	0.205
*PLCG2*	20 (21.5)	3 (17.6)	0.78 (0.20–2.99)	0.720

IPF = idiopathic pulmonary fibrosis. All values are reported as number and percentage. The value represented the number of the patients with rare variants in each gene. P values and odd ratio (OR) with 95% confidence intervals (CIs) were from logistic regression analysis.

## Discussion

In this study of 110 IPF subjects and 60 matched controls, using gene-level association analyses, 226 genes with rare deleterious variants were significantly enriched in the Chinese IPF samples. A large proportion of these genes were first identified in this study. Among the 226 analyzed genes, only *HLA-DRB1* and *HLA-DQB1* genes and their relationships with pulmonary fibrosis were previously analyzed. One genome-wide imputation study reported that two risk alleles, DRB1*15:01 and DQB1*06:02, were associated with fibrotic idiopathic interstitial pneumonias^38^. The association between DRB1*15:01 and IPF was also confirmed in a previous study^39^. Additionally, these two risk alleles were related to the expression of the *DQB1* gene^38^. In addition to *HLA-DRB1* and *HLA-DQB1*, other HLA genes with rare deleterious variants were also significantly associated with IPF in our study, including *HLA-DRB5*, *HLA-A*, *HLA-B*, *HLA-H*, *HLA-G*, *HLA-DQA1* and *HLA-L*. These findings reveal the importance of HLA region for the susceptibility of pulmonary fibrosis^40^.

We also attempted to identify GO category and biological pathways with the input of 226 candidate genes. A total of 36 genes were significantly enriched in the identified GO and KEGG pathways. GO-cellular components showed that these mutated genes were mainly related to vesicle, membrane, extracellular matrix and MHC protein complex. Accordingly, five pathways were highlighted in the KEGG enrichment analyses: ‘antigen processing and presentation’, ‘phagosome’ and ‘cell adhesion molecules (CAMs)’, ‘natural killer cell-mediated cytotoxicity’ and ‘Th1 and Th2 cell differentiation’. Similarly, several previous genome-wide association studies (GWAS) identified common variants in several genes that were relevant to host defense (*ATP11A*, *TOLLIP*^11^) in patients with IPF and cell–cell adhesion (*DSP*, *DPP9*) in patients with fibrotic IIP^10^. Another study by Aquino-Galvez et al.^41^ also indicated that MHC class I chain-related gene A (*MICA*) polymorphisms and abnormal expression of the MICA receptor *NKG2D* were related to the susceptibility of IPF. Besides, Th1/ Th2 cytokine gene polymorphisms were also involved in the etiology and pathogenesis of IPF^42–44^. Although this previous research concentrated on the common variants and our study focused on the rare deleterious variants, these findings suggested that mutated genes involved in the immunity pathway or the cell adhesion pathway might play a role in the risk or susceptibility of IPF.

As for the role of the immunity pathway in IPF, further evidence was obtained from the transcriptomic profiling of cultured lung fibroblasts in IPF patients, showing that 115 downregulated transcripts were enriched in the inflammation and immunity pathways such as defense response to virus, tumor necrosis factor (TNF) mediated signaling pathway, interferon-inducible absent in melanoma2 (AIM2) inflammasome as well as apoptosis^45^. Similarly, another transcriptomic analysis of nasal epithelium in IPF patients indicated that upregulated genes in IPF patients were related to immune response and inflammatory signaling^46^. In turn, the mutated genes enriched in the immunity pathway in our study might partially explain the changes of immunity-related gene expression in these previous studies. However, due to the different directions of immune-related gene expression changes were reported in these two transcriptomic studies, more research is needed to verify our findings and evaluate the function of these genes with rare deleterious variants.

Interestingly, two previous studies (one GWAS^27^ and one WES^28^) also focused on rare protein-altering variants on IPF patients. They found that four genes (*TERT*, *TERC*, *PARN*, *RTEL1*) significantly enriched with candidate rare variants were related to telomere maintenance. And the immune-related and cell adhesion pathways identified in our study may provide new ideas for us to understand the role of rare deleterious protein-altering variants in Chinese IPF.

Immunologic aberrations including immune cells^32,35^, genetic polymorphisms^36^ or gene expression changes^34,37^ were linked to the progression of IPF. Since IPF patients who do not carry the *MUC5B* risk allele have shorter survival from the time of diagnosis than those carrying the risk allele^31^, we also assessed whether immune-related genes with rare deleterious variants were enriched in *MUC5B* non-carriers. However, no significant association was found between them. This might suggest that multiple genetic factors and mechanisms might play roles in IPF progression^27^.

In summary, we evaluate the strengths and limitations of our study. This study was the first to explore rare protein-altering variants using WES in the Chinese IPF population. We aimed to identify genes and biological pathways enriched with rare deleterious variants, which would guide the future genetic and functional studies to elucidate the role of rare variants in the pathogenesis of IPF. Additionally, we included age and gender-matched healthy controls in this study, which was not achieved in the previous related studies^27,28^. However, there are still several limitations to be noted. First, our sample size was small, but we tried to improve our statistical power by using gene-level association analyses instead of single variant association analyses. Second, identified susceptibility genes and pathways have not been replicated and validated in an independent, larger case–control study, therefore, more studies with bigger sample size are needed for future validation analysis.

## Conclusion

In this study, using WES, we identified 226 genes with rare deleterious variants enriched in Chinese IPF patients and drafted a comprehensive mutational landscape of rare protein-coding variants in 36 candidate genes enriched in GO and KEGG pathways. These candidate genes were mainly related to cell adhesion and immune response, which might partially explain changes of gene expression involved in immune-related pathways in IPF. Further validation studies with larger statistical power are needed to verify these findings and identify the underlying functional mechanisms.

## Materials and methods

### Study design and participants

A case–control study consisting of 110 IPF cases and 60 matched controls was conducted at the Peking Union Medical College Hospital (PUMCH) of the Chinese Academy of Medical Sciences and Peking Union Medical College (CAMS & PUMC), Dongcheng District, China. All the subjects were Han Chinese and were enrolled consecutively. All IPF cases had no family history of interstitial lung disease and diagnostic criteria of IPF were based on the American Thoracic Society/European Respiratory Society/Japanese Respiratory Society/Latin American Thoracic Association (ATS/ERS/JRS/ALTA) consensus statement in 2018^47^. Two experienced pulmonologists and one radiologist independently reviewed the clinical and biopsy characteristics and HRCT scans of each patient. Criteria for controls selection included: (1) gender and age-matching, (2) exclusion of pulmonary fibrosis or a family history of interstitial lung disease. Ethical approval of this study was obtained from the Regional Ethics Committee of PUMCH (JS-1127/2016) and procedure of this research conforms to relevant regulations. All participants provided written informed consents. Additionally, demographic information, medical history, family history and other baseline information were collected from each participant.

### Whole-exome sequencing-based approaches for mining of rare deleterious variants in IPF

The process of candidate variant selection and subsequent analysis was shown in Supplementary Fig. S1.

1. Genomic DNA extraction

DNA was extracted from peripheral blood leukocytes using the QIAamp Genomic DNA mini kit (QIAGEN, CA, USA).

2) Library construction and sequencing

A minimum of 1 μg of DNA per sample was used for the DNA library generation using Agilent SureSelect Human All Exon V6 kit (Agilent Technologies, CA, USA) according to the manufacturer’s protocol. First, genomic DNA samples were randomly fragmented by sonication (Covaris, Inc., Woburn, MA, USA) to an average size of 180 ~ 250 bp, followed by end-polishing and A-tailing and ligation of sequencing adaptors. Second, the libraries with special index were hybridized to biotinylated capture probes and they were captured for exome enrichment using magnetic beads with streptomycin, followed by PCR amplification. Third, the concentration of each captured library was accurately determined by quantitative PCR (qPCR) according to the manufacturer's protocol (Agilent Bioanalyzer 2100, Agilent, Santa Clara, CA, USA). Lastly, the qualified DNA libraries were sequenced on Illumina HiSeq X platform (Illumina Inc., San Diego, CA, USA) using 150 bp paired-end reads (PE150).

3) Quality control

For each sample, the following filters were used to select clean reads with high quality: (1) remove the adaptor sequence in reads, (2) remove paired reads if more than 50% of bases were of poor quality (Phred quality ≤ 19) in either one read, (3) remove paired reads if the proportion of uncertain bases was over 10% in either one read.

4) Detection and filtering of genomic alterations

Whole-exome valid sequencing reads were mapped to the reference human genome (GRCh37/hg19), using Burrows-Wheeler Aligner (BWA) software to generate original BAM files. Then, these files were sorted and realigned by SAMtools to compute the sequence coverage and depth^48^. Duplicate reads were marked and removed using the Picard suite. Finally, single-nucleotide variants (SNVs) and insertions and deletions (indels) were called with GATK^49^ and annotated by ANNOVAR^50^. Following filters were set to identify candidate variants: (1) keep mutations with coverage ≥ 10 × , unless the variant had high impact (e.g.: stop gain, stop loss, frameshift), which required coverage ≥ 5 × ; (2) remove variants with mutant allele frequency (MAF) ≥ 0.01 in East Asian (EAS) population in the Exome Aggregation Consortium (ExAC) database, 1000 Genomes or Genome Aggregation Database (GnomAD); (3) variations in the exonic or splicing region (10 bp upstream and downstream of splicing sites); (4) keep variants if the functional predictions by SIFT (dbNSFP version 3.0, D: Deleterious), PolyPhen-2 (dbNSFP version 3.0, D: Probably damaging), MutationTaster (dbNSFP version 3.0, A: Disease causing automatic or D: Disease causing) and CADD (dbNSFP version 3.0, PHRED-like score > 20) all indicated the SNV was not benign or if it had high impact (e.g.: stop gain, stop loss, frameshift).

### Population structure analysis

Principal component analysis (PCA) for all samples was performed using software GCTA^51^ (version 1.93.2beta, http://cnsgenomics.com/software/gcta), including all candidate variants after multi-step filtering. Significant PCs were inferred using Tracy-Widom statistics (P value < 0.05)^52,53^. The results showed that no significant PC was found. There was no obvious difference between the case group and the control group (Supplementary Fig. S2). This confirmed that all the samples were Han Chinese and there was no population stratification.

### Gene-based collapsing analysis

Single-variant tests are less capable of identifying rare variants than common variants^54^. To make them more capable of detecting association of rare variants that cluster in the individual genes, gene-level collapsing analysis was performed using SKAT^55,56^. Only 10,333 candidate variants were included for gene-based association analysis. Candidate genes enriched with rare deleterious variants were identified if P value (corrected by Benjamini–Hochberg false discovery rate (FDR)) from SKAT was less than 0.05. Considering the complexity of HLA region, we tried to use another common collapsing analysis^28,57,58^ to further determine the relationship between HLA genes and IPF. The detailed steps were as follows: for each of the candidate HLA genes (*HLA-A*, *HLA-B*, *HLA-DQA1*, *HLA-DQB1*, *HLA-DRB1*, *HLA-DRB5*, *HLA-G*, *HLA-H*, *HLA-L*) selected from SKAT, we assigned an indicator variable (1/0 states) to each individual based on the presence of at least one candidate variant in that gene (state 1) or no candidate variants in that gene (state 0). Then we used the two-tailed Fisher’s exact test for each gene to compare the rate of case subjects carrying a candidate variant compared with the rate of control subjects and P value was corrected by FDR.

### Enrichment analysis and pathway analysis

To investigate the biological relevance of the candidate genes, gene ontology (GO) enrichment analysis was performed to categorize the function of these genes into three classes: ‘biological process’, ‘cellular components’, and ‘molecular function’. Additionally, the Kyoto Encyclopedia of Genes and Genomes (KEGG) database^59^ (http://www.genome.jp/kegg/pathway.html) was used to identify pathways that were enriched with candidate genes in IPF. P value was adjusted by FDR.

### *MUC5B* rs35705950 genotyping

*MUC5B* rs35705950 within the promoter region of the *MUC5B* gene (chr11:g.1241221G.T, NCBI Build 37) was genotyped using the Sanger sequencing after the PCR amplification. Sanger sequencing was performed using the Big Dye v.3.1 terminator cycle sequencing kit and an Applied Biosystems 3730xl capillary sequencer (Applied Biosystems, CA, USA). Primers were as follows, forward, TGGCCAGAATGAGGGACAGT; reverse, GACGTCAAGGCCACAGCTAT. The risk allele of *MUC5B* 35705950 was T while the non-risk allele was G.

### Statistical analysis

Statistical analysis was performed using SPSS software version 24.0 for Windows (SPSS Inc., Chicago, IL, USA) and R statistical software (version 3.51). Two-tailed P (or P.adjusted) < 0.05 was considered statistically significant. Results of continuous variables were reported as mean ± standard deviation (SD), while categorical variables were reported as a number with a percentage. Comparison of basic characteristics between two groups was done by using the Student’s t-test for continuous variables which fulfilled homogeneity of variance and by using a chi-square test for categorical variables. Logistic regression was used to assess associations between rs35705950 SNP and genes enriched in the immunity pathway. Results of logistic regression are presented as odd ratio (OR) with 95% confidence intervals (CIs).

## Supplementary Information

Supplementary Information.

## Data Availability

The datasets generated during and/or analyzed during the current study are available from the corresponding author on reasonable request.
