# Supplementary material for: Whole-exome sequencing identifies susceptibility genes and pathways for idiopathic pulmonary fibrosis in the Chinese population
Source: Sci Rep. 2021 Jan 14;11:1443. doi: 10.1038/s41598-020-80944-4 (PMC7809470; doi:10.1038/s41598-020-80944-4)
Supplement: Supplementary file 1 — Supplementary Information. [file 41598_2020_80944_MOESM1_ESM.pdf]

Whole-exome sequencing identifies susceptibility genes and pathways for idiopathic pulmonary fibrosis in the Chinese population

Chuling Fang[1], Hui Huang[1], Yujia Feng[2], Qian Zhang[1], Na Wang[1], Xiaoyan Jing[1], Jian Guo[1], Martin Ferianc[3], Zuojun Xu[1]*

[1]Department of Respiratory and Critical Medicine, Peking Union Medical College Hospital, Chinese Academy of Medical Sciences & Peking Union Medical College, Beijing, China.

[2]The Bioinformatics department, Digital China Health Technologies Co., Ltd, Beijing, china.

[3]Electronic and Electrical Engineering Department, University College London, London, UK.

* E-mail: xuzj@hotmail.com.

Fig S1 Flowchart of the study design for the rare variant analysis.

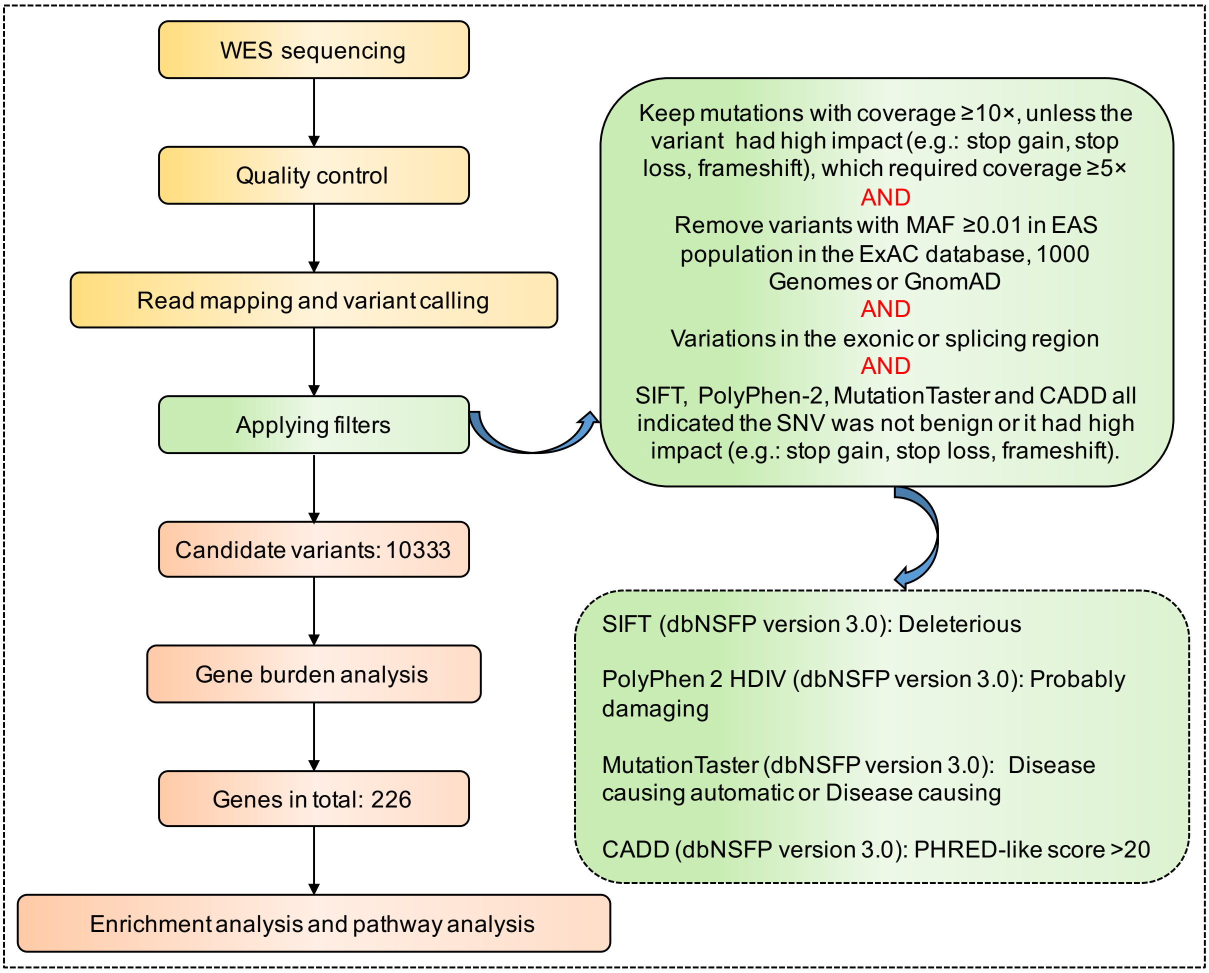

Four filters used to identify deleterious variants and the thresholds of four software (SIFT, PolyPhen-2, MutationTaster and CADD) used to identify deleterious variants were shown in green box above. MAF=mutant allele frequency, EAS=East Asian, ExAC=Exome Aggregation Consortium,

GnomAD=Genome Aggregation Database.

Fig S2 Principal Component Analysis of 110 IPF samples and 60 healthy control samples.

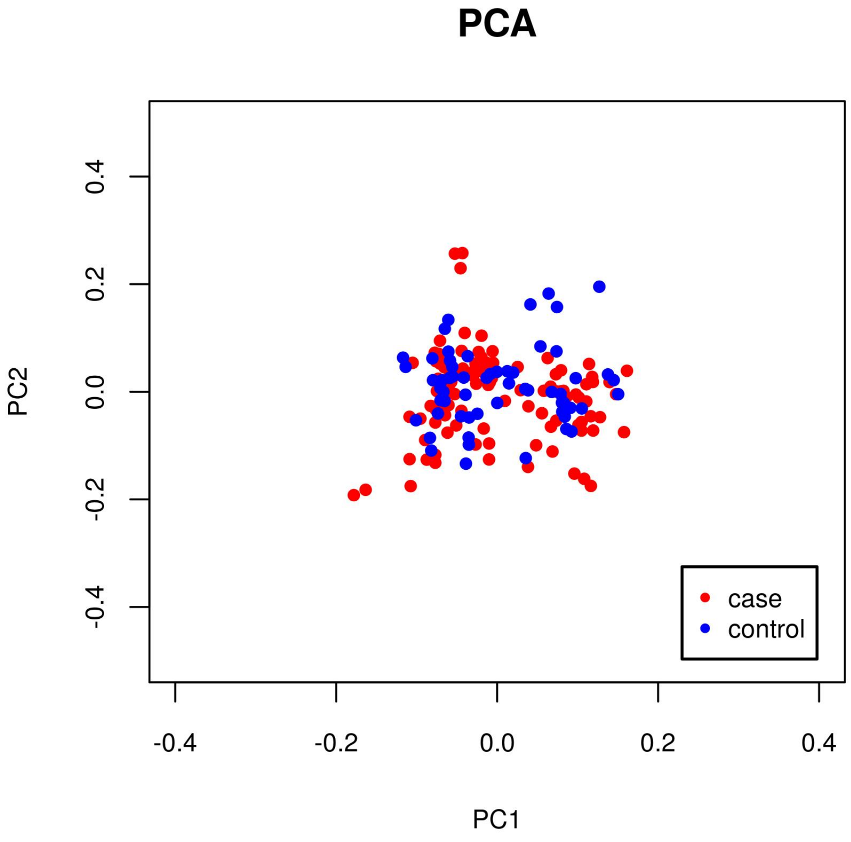

Fig S3: Coverage of variants identified in HLA regions.

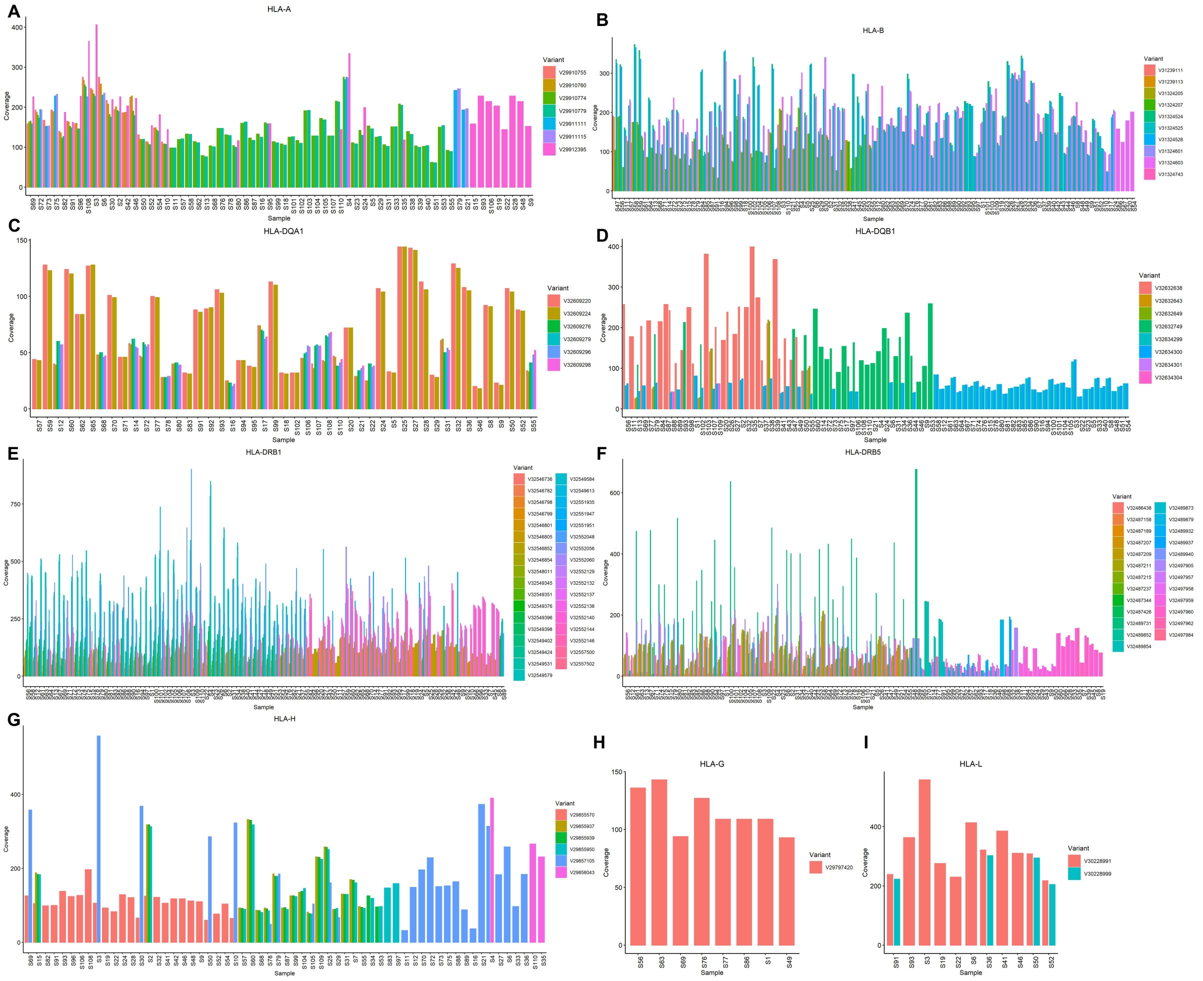

Table S1 226 candidate genes enriched with rare deleterious variants.

Gene	Variant_count	P-FDR from SKAT
DDX11L1	1	0.015737394
OR4F5	1	0.046780623
KLHL17	1	0.000952768
RNF207	2	0.041229025
PRAMEF1	3	6.28E-14
PRAMEF2	4	0.022339276
MST1L	11	4.24E-14
AX748283	4	0.00019935

BC038455	2	0.00250716
UBXN11	7	0.000488736
KIAA1522	4	0.004535233
TIE1	5	3.18E-05
TTC39A	2	7.70E-06
FAM72D	1	1.61E-14
LOC100288142	2	0
PDE4DIP	5	2.47E-05
SEC22B	3	0.014669238
NBPF12	1	1.14E-06
NBPF14	2	0
NBPF16	1	0
HRNR	10	3.61E-06
NES	4	0.000175388
HSPA6	6	0.008187631
KIAA0040	4	0.023411563
PTPRVP	5	0.013689552
CEP170	2	0.0049815
OR2T3	1	0.046780623
OR2T5	1	1.10E-09
OR2T29	1	0.001969812
BC046483	5	3.06E-05
PTCHD3	3	0.042739341
AGAP4	2	0.005652204
FAM178A	1	0.016902956
FANK1	1	0.046780623
PIDD	1	0.008161304
C11orf40	4	0.008633502
OR51A4	3	0.015471737
LINC00294	1	0.00448289
MDK	13	2.07E-06
FOLH1	1	0.046780623

METTL12	3	0.042041974
SLC22A9	1	0.015535744
ESRRA	5	2.96E-11
TRIM64B	2	0.047976413
CASP5	2	0.04837382
AK130852	10	0.015737394
LRTM2	1	0.038442492
PHC1	1	0.000201375
TAS2R46	2	0.011392102
PRB3	4	7.16E-09
PRB4	2	0.028334864
PRB1	3	0.000246804
SLCO1A2	1	0.046780623
KRT6B	2	4.24E-14
LOC283335	1	7.99E-10
STAT2	1	0.046780623
BC033961	1	0.005627372
KIAA1033	1	0.046780623
GATC	1	0.038442492
CAMKK2	5	0.036283865
KNTC1	5	0.046459129
SKA3	4	0.01867057
PARP4	4	1.84E-08
PABPC3	15	1.84E-09
C14orf23	4	0.018754551
WDR89	8	0.008225472
DKFZp686O16217	2	0.009454911
ELK2AP	1	9.00E-05
FLJ00382	1	0.00115614
GOLGA6L6	3	0.00031415
LOC283710	2	0.000172033
CHRNA7	3	0.011065819

MAPKBP1	4	0.025202021
SORD	3	0.028334864
CSK	1	0.044777337
CSPG4	2	7.17E-06
DNM1P41	3	0.036629748
PCSK6	2	0.00115614
WASH3P	2	0.016387193
SSTR5-AS1	4	0.004535233
TSR3	2	0.048466823
GNPTG	1	0.038442492
NMRAL1	1	0.038442492
AK055785	2	5.22E-08
OTOA	2	0.011519765
LOC653786	1	0.0049815
ZNF629	1	0.038442492
CES1	5	0.003593626
PDPR	4	0.000203198
NPIPL2	2	0
CLEC18B	5	0.02139066
PLCG2	3	0.042041974
C16orf85	2	0.018911871
AK055272	3	0.001218903
AK302511	1	0.036283865
CNTROB	2	0.048466823
MEIS3P1	1	3.18E-05
TOM1L2	3	0.009237796
C17orf103	1	9.37E-06
KCNJ12	4	4.89E-07
GOSR1	4	4.30E-05
COPZ2	2	3.45E-05
BC033456	2	4.63E-08
USP32	1	0.028334864

TBC1D3P2	2	2.47E-10
CCDC40	3	0.00135667
AK127919	1	3.18E-05
SOGA2	8	0.001314571
KIAA1468	2	0.002866685
OR4F17	1	3.72E-05
REEP6	1	0.046780623
ATP8B3	5	0.000892129
KRI1	3	0.038442492
ANKLE1	7	7.66E-06
B3GNT3	5	3.27E-05
ZNF626	2	0.018286361
RHPN2	1	4.01E-06
U2AF1L4	6	0.006639426
LOC644189	2	8.45E-06
CD177	2	3.64E-06
KLC3	5	0.000100495
MYBPC2	4	0.048113376
SIGLEC10	2	0.001619871
ZNF880	13	0.031549467
ZNF83	2	0.005301149
LILRA6	4	3.27E-05
KIR2DL1	3	3.77E-13
KIR2DS4	1	0.011649469
MGC2752	1	3.03E-07
FAM136A	2	4.76E-08
RGPD1	3	1.23E-05
VWA3B	3	0.029704755
GCC2	1	2.71E-06
ANAPC1	3	0.001666354
RGPD5	2	0.000575101
HS6ST1	2	0

SMPD4	5	0.008187631
POTEE	5	0
PLA2R1	1	0.046459129
ITGA6	3	0.043859594
PRKRA	2	0.00019935
CCDC150	4	0.000913164
ANKRD44	1	0.008633502
FTCDNL1	2	0.000355721
PRR21	2	0.013903322
AQP12A	2	0.008187631
BC101234	3	2.60E-09
LOC339593	1	0.01279849
BFSP1	3	0.00073135
RIN2	6	0.042739341
LAMA5	7	0.029704755
BAGE3	1	0.009431036
POM121L7	3	5.62E-12
BCR	2	3.15E-09
AK026502	2	6.28E-14
TCN2	2	0.046780623
SYN2	3	0.000188538
VENTXP7	3	0.000493286
GOLGA4	2	0.046780623
MST1	5	0.004535233
LINC00636	2	2.28E-06
SDHAP2	2	9.79E-08
MUC4	19	0.00870228
ABCA11P	1	0.021026873
OTOP1	3	4.89E-10
USP17L10	2	9.52E-14
USP17L11	4	0.009040961
USP17L20	1	0.028334864

LPHN3	1	0.016902956
SLC9B1	4	0
DCHS2	7	7.22E-05
ENPP6	1	0.016902956
SDHA	3	5.91E-05
C9	1	0.038442492
LOC100272216	3	0
BTF3	1	0.036629748
ERAP1	1	0.016902956
PCDHB16	1	0.046780623
GPRIN1	2	0.020850546
LOC100132062	1	6.04E-06
FAM8A1	2	0.00297184
HLA-G	1	0.038237787
HLA-H	7	2.17E-05
AK309533	6	7.51E-06
HLA-A	7	1.49E-12
HLA-L	2	0.018046635
HLA-B	11	0
TNXB	4	0.000128876
HLA-DRB5	26	0
HLA-DRB1	36	0
HLA-DQA1	6	2.26E-08
HLA-DQB1	8	0
SYNGAP1	5	0.001635482
C6orf57	1	0.044777337
ANKRD6	1	0.021026873
GPR126	4	2.02E-05
FNDC1	4	0.046780623
LINC00473	1	0.016632759
TCP10L2	1	0.000145558
LFNG	2	0.00038938

ZNF890P	2	0.007840158
LOC402470	1	0.009993988
ZNF733P	1	0.012159401
ZNF107	5	0.02238969
MUC17	9	4.33E-06
RABL5	4	0.000166946
POLR2J3	2	0
RASA4	4	0.018995754
FAM185A	1	3.18E-05
FLJ45340	2	3.18E-05
FAM115C	2	1.22E-05
ARHGEF5	7	0.005992449
AGAP3	7	3.98E-10
KMT2C	8	1.24E-13
FAM86B1	2	1.95E-11
FAM86B2	3	0.000493724
LOC729732	2	0.046780623
NUDT18	3	0.014594909
CPNE3	2	0.040883428
FOXD4L3	2	0.003288371
OR13C5	3	6.83E-09
CEL	2	0.000142615
OLFM1	1	0.028334864
AK096249	2	0.028334864
RBMX	1	0.001846669
CD24	4	0.01876791

Variant count: the number of deleterious variants detected in each gene. P-FDR: P values were from SKAT and corrected by false discovery rate (FDR).
